# Using structural equation modeling for network meta-analysis

**DOI:** 10.1186/s12874-017-0390-9

**Published:** 2017-07-14

**Authors:** Yu-Kang Tu, Yun-Chun Wu

**Affiliations:** Department of Public Health and Institute of Epidemiology and Preventive Medicine, College of Public Health, National Taiwan University, Taipei, Taiwan

**Keywords:** Randomized controlled trials, Network meta-analysis, Mixed treatments comparisons, Structural equation modeling, Generalized linear mixed models, Multivariate meta-analysis

## Abstract

**Background:**

Network meta-analysis overcomes the limitations of traditional pair-wise meta-analysis by incorporating all available evidence into a general statistical framework for simultaneous comparisons of several treatments. Currently, network meta-analyses are undertaken either within the Bayesian hierarchical linear models or frequentist generalized linear mixed models. Structural equation modeling (SEM) is a statistical method originally developed for modeling causal relations among observed and latent variables. As random effect is explicitly modeled as a latent variable in SEM, it is very flexible for analysts to specify complex random effect structure and to make linear and nonlinear constraints on parameters. The aim of this article is to show how to undertake a network meta-analysis within the statistical framework of SEM.

**Methods:**

We used an example dataset to demonstrate the standard fixed and random effect network meta-analysis models can be easily implemented in SEM. It contains results of 26 studies that directly compared three treatment groups A, B and C for prevention of first bleeding in patients with liver cirrhosis. We also showed that a new approach to network meta-analysis based on the technique of unrestricted weighted least squares (UWLS) method can also be undertaken using SEM.

**Results:**

For both the fixed and random effect network meta-analysis, SEM yielded similar coefficients and confidence intervals to those reported in the previous literature. The point estimates of two UWLS models were identical to those in the fixed effect model but the confidence intervals were greater. This is consistent with results from the traditional pairwise meta-analyses. Comparing to UWLS model with common variance adjusted factor, UWLS model with unique variance adjusted factor has greater confidence intervals when the heterogeneity was larger in the pairwise comparison. The UWLS model with unique variance adjusted factor reflects the difference in heterogeneity within each comparison.

**Conclusion:**

SEM provides a very flexible framework for univariate and multivariate meta-analysis, and its potential as a powerful tool for advanced meta-analysis is still to be explored.

**Electronic supplementary material:**

The online version of this article (doi:10.1186/s12874-017-0390-9) contains supplementary material, which is available to authorized users.

## Background

Meta-analysis is a very important methodological tool for evidence synthesis [1]. Traditional meta-analysis compares outcomes of two groups directly using data from studies in which the difference in the results between these two groups were tested. When more than two groups are to be compared, multiple pairwise meta-analyses need to be undertaken. When two of those groups have never been compared directly by any study, it becomes impossible to undertake the traditional meta-analysis for them. Even if each pair of those groups have been compared directly, different pairwise comparisons involve different studies using different evidence bases in their comparisons, and the results may not be consistent. For instance, in three pairwise comparisons for groups A, B, and C, pairwise meta-analyses may show A is better than B, B is better than C, but A is not better than C. The limitations of the traditional approach to comparing multiple groups have been documented extensively [2–6].

One recent development in meta-analysis methodology to resolve those issues is network meta-analysis for comparisons of multiple treatment groups [7–15]. Network meta-analysis incorporates all available evidence into a general statistical framework to yield consistent results for comparisons of all available treatments. Whilst the idea of indirect comparisons for treatments that had not been tested directly was first proposed in 1990s [16, 17], Lumley coined the term network meta-analysis and proposed a linear mixed model approach to comparisons of multiple treatments within the same statistical model [7]. Later, Lu and Ades developed a sophisticated Bayesian hierarchical model, providing a flexible statistical framework to take into account the complexity in the data structure within multi-arm trials [11]. Their statistical approach, widely known as mixed treatments comparison or Bayesian network meta-analysis, has been a popular approach to comparisons of multiple treatments [5, 12, 18, 19].

Several recent articles looked further into the complexity in modeling multiple treatments comparisons with an attempt to implement Lu and Ades’s approach within the generalized linear mixed modelling framework [8, 12, 19, 20], to make network meta-analysis more accessible to clinicians and meta-analysts who are not familiar with Bayesian statistics. However, it is quite a challenging task to develop a formal statistical model for undertaking multiple treatment comparisons [9, 11, 18, 19, 21–25]. Two specific issues arise from implementing Lu & Ades’s Bayesian model into generalized linear mixed model: First, Lu and Ades’s approach uses the contrast between two treatment groups, such as log odds ratio or differences in means, as the outcome, and consequently, treatment contrasts between any pair of treatments within a multi-arm study are not independent; their correlations therefore need to be taken into account in the model [26, 27]. Secondly, as the random effect structure for those treatment contrasts to address the heterogeneity becomes increasingly complex when the number of treatments involved in a network meta-analysis increases, specifying the random effect structure with treatment contrasts as the outcomes is not a simple task [12].

Structural equation modeling (SEM) is a statistical method originally developed for modeling causal relations among observed and latent variables. It can also be used to analyze longitudinal data and its results have been shown to be equivalent to those from multilevel modeling. Recent developments in SEM extend its application to multilevel data and non-continuous dependent variables. Consequently, generalized linear mixed modeling can now be undertaken within SEM framework. As random effect is explicitly modeled as a latent variable in SEM, it is very flexible for analysts to specify complex random effect structure and to make linear and nonlinear constraints on parameters. Those advantages have been shown to be very useful for undertaking multivariate meta-analysis within SEM [28–30]. In our previous studies, we have shown how to undertake network meta-analysis by means of generalized linear mixed modelling [25, 31–33]. In this article, we attempt to develop a SEM approach to network meta-analysis based on the Lu & Ades’s model. This article is organized as follows: we first briefly review the Lu & Ades model and show how it can be implemented within generalized linear mixed models using treatment contrasts as the outcome. We then use an example to show how SEM can be used to undertake a network meta-analysis for the fixed and random effect network meta-analysis and how the weighting for each study can be taken into account. Finally, we demonstrate how a new approach to network meta-analysis, namely the unrestricted weight least squares (UWLS) method, can be implemented in SEM.

## Methods

There are two models for network meta-analysis: fixed effect model assumes that treatment effects are common across studies, and random effect model assumes that treatment effects are heterogeneous across studies.

### Fixed effect model for network meta-analysis

The fixed effect network meta-analysis for multiple treatment comparisons based on Lu and Ades’s approach can be specified as:

1 $$ {g\left({\hat{y}}_{k\cdot j}\right)=\eta}_{k\cdot i}=\Big\{\begin{array}{ll}{\mu}_{b\cdot j}&, b= A, B, C,\dots \kern0.75em \mathrm{if}\  k= b\\ {}{\mu}_{b\cdot j}+{d}_{b k}={\mu}_{b\cdot j}+{d}_{Ak}-{d}_{Ab}&, k= B, C, D,\dots \kern0.75em \mathrm{if}\  k\ {\mathrm{is}}^{\prime }{\mathrm{after}}^{\prime } B\end{array}\operatorname{} $$

where treatments are coded as A, B, C,..., K, and K is the number of treatments to be compared within the network. The ${\hat{y}}_{k\cdot j}$ is the expected value for *y*_*k*_._*j*_, which is the observed outcome for treatment *k* in study *j*. The *g*() is a link function for the model to transform the ${\hat{y}}_{k\cdot j}$ to *η*_*k*_._*j*_, which is the expected value given by the model for arm *k* in study *j*, and *μ*_*b*_._*j*_ is the baseline treatment effect in trial *j*. The difference between the other treatment *k* and treatment *b* in the same trial will be estimated by expressing them in terms of effects relative to the treatment A, which is the global baseline treatment within the whole network. Due to identification reason and its interpretation as the effect of treatment A compared to itself, *d*_*AA*_ is fixed at 0, and Lu and Ades called *d*_*AB*_ to *d*_*Ak*_ the basic parameters. The advantage of expressing all treatment comparisons as the relations between basic parameters is that the number of pairwise comparisons to be estimated for a network meta-analysis involving *k* treatments is reduced to *k* – 1 for the fixed effect [34].

### Random effect model for network meta-analysis

For the random effect network meta-analysis, *d*_*bk*_ in Eq. (1) is replaced by *δ*_*kb*_._*j*_, the trial-specific effect of treatment *k* relative to trial-specific baseline treatment *b*, and the equation is given as:

2 $$ {g\left({\hat{y}}_{k\cdot j}\right)=\eta}_{k\cdot i}=\Big\{\begin{array}{ll}{\mu}_{b\cdot j}&, b= A, B, C,\dots \kern0.5em \mathrm{if}\  k= b\\ {}{\mu}_{b\cdot j}+{\delta}_{b k\cdot j}&, k= B, C, D,\dots \kern0.5em \mathrm{if}\  k\ {\mathrm{is}}^{\prime }{\mathrm{after}}^{\prime } B\end{array}\operatorname{} $$

These trial-specific effects are then drawn from a normal distribution: ${\delta}_{bk\cdot j} \sim N\left({d}_{bk},{\tau}_{bk}^2\right)$. Then, *d*_*bk*_ is expressed in terms of the basic parameters: *d*_*bk*_ = *d*_*Ak*_ − *d*_*Ab*_, with *d*_*AA*_ being fixed at 0 [9, 12, 35]. Note that although the model in Eq. (2) uses data from each treatment arm of a study, it selects one treatment within each study as the trial-specific baseline treatment to estimate the treatment contrast between this baseline treatment and other treatments within the same study. When a study consists of more than two treatment arms, it will contribute more than one treatment contrast, and these treatment contrasts are not independent. Therefore, *δ*_*bk*_._*j*_ will follow a multivariate normal distribution. For instance, suppose study 1 compares treatment B, C and D, and *δ*_*BC*__.1_ and *δ*_*BD*__.1_ in Eq. (2) for this study will then follow bivariate normal distribution:

$$ \left(\begin{array}{c}{\delta}_{BC\cdot 1}\\ {}{\delta}_{BD\cdot 1}\end{array}\right) \sim M V N\left(\begin{array}{c}{d}_{BC}\\ {}{d}_{BD}\end{array},\left[\begin{array}{cc}{\tau}_{BC}^2& cv\\ {} cv& {\tau}_{BD}^2\end{array}\right]\right) $$

Where *cv* is the covariance between ${\tau}_{BC}^2$ and ${\tau}_{BD}^2$. In the Lu & Ades approach, all the random effect variances are constrained to be equal, i.e. ${\tau}_{BC}^2={\tau}_{BD}^2={\tau}^2$, and *cv* is $\frac{1}{2}{\tau}^2$, i.e. the correlation between random effects is 0.5 [11].

### Contrast-based model

To implement the treatment contrasts model in Eqs. (1) and (2) into general or generalized linear mixed model, we can either use the contrast-based approach [36], where treatment contrasts are derived from each study before undertaking network analysis, or use the arm-based approach [25, 37], where data from each arm is used directly. For the contrast-based approach, the dependency of treatment contrasts within a multi-arm trial needs to be taken into account in the model. As taking into account this dependency is not straightforward in most software packages, data transformation using some matrix algebra techniques can be used to create an independent dataset [25, 28, 31]. In the contrast-based fixed effect model shown in Eq. (1), effect size summary odds ratio or risk ratio, needs to be transformed into natural log odds ratio or risk ratio, which behaves approximately as a normal, and the model can now be written as:

3 $$ \begin{array}{l}{\varDelta}_{i\cdot j}={\sum}_{k= B}^K{b}_{Ak}{t}_{Ak}+{v}_{i\cdot j}\\ {}{v}_{i\cdot j} \sim N\ \left(0,{\sigma}_{i\cdot j}^2\right)\end{array}, $$

where *Δ*_*i*_._*j*_ is the effect size summary of the *i* ^th^ treatment contrast in study *j* such as difference in means or log odds ratio, *t*_A*k*_ is the contrast coding dummy variable for treatment contrast A versus *k* for *k* = B to K, *b*_AB_ to *b*_AK_ are regression coefficients for treatment contrasts A versus B to A versus K in the network, and ${\sigma}_{i\cdot j}^2$ is the known variance of *Δ*_*i*_._*j*_. The vector **b** for regression coefficients can be obtained by [38]:

4 $$ \mathbf{b}={\left({\mathbf{X}}^{\mathbf{T}}{\mathbf{V}}^{-1}\mathbf{X}\right)}^{-1}{\mathbf{X}}^{\mathbf{T}}{\mathbf{V}}^{-1}\boldsymbol{\Delta}, $$

where the matrix **X** contains all the covariates *t*_AB_, *t*_AC_,…, and *t*_AK_, **X** ^**T**^ is the transposed **X**, **Δ** is the vector of *Δ*_*i*_._*j*_, and **V** ^−1^is the inverse of the block-diagonal matrix **V**:

$$ \mathbf{V}=\left[\begin{array}{cccc}\hfill {\mathbf{V}}_{\mathbf{1}}\hfill & \hfill \mathbf{0}\hfill & \hfill \mathbf{0}\hfill & \hfill \mathbf{0}\hfill \\ {}\hfill \mathbf{0}\hfill & \hfill {\mathbf{V}}_{\mathbf{2}}\hfill & \hfill \mathbf{0}\hfill & \hfill \mathbf{0}\hfill \\ {}\hfill \vdots \hfill & \hfill \vdots \hfill & \hfill \ddots \hfill & \hfill \vdots \hfill \\ {}\hfill \mathbf{0}\hfill & \hfill \mathbf{0}\hfill & \hfill \mathbf{0}\hfill & \hfill {\mathbf{V}}_J\hfill \end{array}\right] $$

The diagonal elements in **V** are **V**_*j*_, *j* = 1 to *J*, the variance-covariance matrix of *v*_*i*_._*j*_ in Eq. (3). **V**_*j*_ is a scalar if study *j* is a two-arm study and a matrix if study *j* is a multi-arm study. Cheung proposed to use Cholesky decomposition to decompose **V** ^−1^ = **LL** ^**T**^, where **L** is a lower triangular matrix and **L** ^**T**^ is the transpose of **L** [28]. We can pre-multiply **X** and **Δ** by **L** ^**T**^ to obtain the transformed matrix $\overset{\sim }{\mathbf{X}}={\mathbf{L}}^{\mathbf{T}}\mathbf{X}$ and the transformed vector $\overset{\sim }{\boldsymbol{\Delta}}={\mathbf{L}}^{\mathbf{T}}\boldsymbol{\Delta}$. So Eq. (4) can be re-written as:

5 $$ \mathbf{b}={\left({\overset{\sim }{\mathbf{X}}}^{\mathbf{T}}\overset{\sim }{\mathbf{X}}\right)}^{-1}{\overset{\sim }{\mathbf{X}}}^{\mathbf{T}}\overset{\sim }{\boldsymbol{\Delta}}. $$

Under this transformation, the impact of *v*_*i*_._*j*_ in Eq. (3) has been absorbed into $\overset{\sim }{\mathbf{X}}$ and $\overset{\sim }{\boldsymbol{\Delta}}$, so Eq. (3) can be re-written as an ordinary least squares model:

$$ \begin{array}{l}{\overset{\sim }{\varDelta}}_{i\cdot j}={\sum}_{k= B}^K{b}_{Ak}{x}_{Ak}+{e}_{i\cdot j}\\ {}{e}_{i\cdot j} \sim N\ \left(0,1\right)\end{array} $$

where ${\overset{\sim }{\varDelta}}_{i\cdot j}$ is the transformed *Δ*_*i*_._*j*_, and *x*_A*k*_ is the transformed *t*_A*k*_ in Eq. (3).

### Example data: sclerotherapy

The example dataset contains results of 26 studies that directly compared three treatment groups A, B and C for prevention of first bleeding in patients with liver cirrhosis [39]: A was the control group, B was sclerotherapy, and C was the use of beta-blocker. The whole dataset can be found in the Additional file 1. Among the 26 study, two are three-arm trials, and seven compared A to C and 17 compared A to B. Throughout the analysis in this article, treatment A was chosen as the global baseline treatment.

As the outcome is a binary variable, the difference in the outcome between any two treatments may be expressed as odds ratio or risk ratio, but to undertake a trial-based approach, we need to take a natural log transformation of odds ratio or risk ratio. Here, we used log odds ratio as the effect size measure. For the three-arm trials, we calculated two treatment contrasts, A vs B and A vs C, and the covariance between the two correlated treatment contrasts is the variance of log odds ratio for treatment A. The regression model for the fixed effect network meta-analysis is therefore written as:

6 $$ \begin{array}{l} \ln {OR}_{i\cdot j}={b}_{AB}{t}_{AB}+{b}_{AC}{t}_{AC}+{v}_{i\cdot j}\\ {}{v}_{i\cdot j} \sim N\ \left(0,{\sigma}_{i\cdot j}^2\right)\end{array} $$

where ln*OR*_*i*_._*j*_ is the *i* ^th^ log odds ratio for study *j*, ${\sigma}_{i\cdot j}^2$ is the variance of ln*OR*_*i*_._*j*_, *t*_AB_ is a dummy variable where treatment contrast for A versus B is denoted 1 and contrast for A versus C denoted 0, and *t*_AC_ a dummy variable where treatment contrast for A versus C is denoted 1 and contrast for A versus B denoted 0. Note that if there are trials that compared B to C, *t*_AB_ would coded −1 and *t*_AC_ coded 1 for those trials [25]. The regression coefficient *b*_AB_ and *b*_AC_ in Eq. (6) cannot be directly estimated in SEM, because ln*OR*_*i*_._*j*_ are not independent in the three-arm trials; but *b*_AB_ and *b*_AC_ can be obtained by transforming ln*OR*_*i*_._*j*_, *t*_AB_ and *t*_AC_ using the procedure described in the previous section. For the random effect model, *b*_*AB* . *j*_ and *b*_AC . *j*_ in Eq. (6) are replaced with ${b}_{AB. j}^{\ast }$ and ${b}_{AC. j}^{\ast }$, which are assumed to follow a bivariate normal distribution:

7 $$ \left(\begin{array}{c}{b}_{AB. j}^{\ast}\\ {}{b}_{AC. j}^{\ast}\end{array}\right) \sim MVN\left(\begin{array}{c}{\beta}_{AB}\\ {}{\beta}_{AC}\end{array},\left[\begin{array}{cc}{\tau}^2& \frac{1}{2}{\tau}^2\\ {}\frac{1}{2}{\tau}^2& {\tau}^2\end{array}\right]\right), $$

where the *β*_AB_ and *β*_AC_ are the average treatment effect difference between A and B and between A and C, respectively; and *τ* ^2^ is the treatment effect variability across studies.

### Contrast-based SEM network meta-analysis

SEM is a multivariate statistical analysis technique that is a combination of factor analysis and multiple regression analysis [40]. Many traditional statistical methods such as analysis of variance, regression analysis, and factor analysis can therefore be considered as special models of SEM. Traditional SEM requires that the outcome variables and the latent constructs have to be continuous, but with new development of SEM theory and software packages, these are no longer limitations of SEM. As a result, generalized linear mixed models and SEM can now be considered generalized latent variable models [41]. The main difference between SEM and generalized linear mixed models is that random effects are explicitly specified as latent variables in SEM and relationships between observed/latent variable are explicitly specified as causal or non-causal. A comprehensive overview of SEM is beyond the scope of this article, and readers can find an in-depth discussion of applications of SEM to univariate and multivariate meta-analyses in a series of articles and a textbook [28–30, 42–45].

Although network meta-analysis can now be undertaken within the statistical framework of generalized linear mixed models, we feel integrating network meta-analysis into SEM framework has several advantages: first, network meta-analysis can be visualized in SEM, and this can be useful for understanding the complexity of the model, especially when analysts wish to look into the role of potential effect modifiers or moderators in the comparisons of multiple treatments by undertaking meta-regression [46]. Secondly, SEM software packages are more flexible in making constraints on model parameters such as regression coefficients, variances and covariances, because random effects are explicitly modelled as latent variables. Thirdly, SEM is a primary research tool for social scientists, but they are less familiar with network meta-analysis, which is becoming more and more popular in biological and medical research. Therefore, integrating network meta-analysis into SEM framework will bring network meta-analysis to attentions of greater audiences [45].

### UWLS for meta-analysis

Recently, a new approach has been proposed for meta-analysis, which differs from the standard fixed or random effect models [47, 48]. The standard fixed effect meta-analysis for pairwise comparisons is just weight least squares regression and can be written as:

8 $$ {\varDelta}_j=\mu +{v}_j $$

where *Δ*_*j*_ may be the log odds ratio or difference in means between two treatments, *v*_*j*_ is the standard error of *Δ*_*j*_ and ${v}_j \sim N\left(0,{\sigma}_j^2\right)$, where ${\sigma}_j^2$ is the variance of *Δ*_*j*_. In the (UWLS approach, the variance of *v*_*j*_ is in proportion to the variance of *Δ*_*j*_, i.e. ${v}_j \sim N\left(0,\phi {\sigma}_j^2\right)$. The introduction of variance adjustment factor *ϕ* to Eq. (8) will not affect the point estimate for *μ*, but its standard error will be affected: when *ϕ* is larger than 1, the confidence interval for *μ* will be greater than that given by the standard fixed effect model, but in contrast, when *ϕ* is smaller than 1, the confidence interval for *μ* will become smaller. According to recent studies [47, 49], UWLS approach provides satisfactory estimates and confidence intervals that are comparable to random effects when there is no publication bias and identical to fixed-effect meta-analysis when there is no heterogeneity.

### UWLS for network meta-analysis

In network meta-analysis, the numbers of studies involved in pairwise comparisons are usually quite different, and the degree of heterogeneity within each pairwise comparison also varies. Therefore, network meta-analysis usually uses random effect model to take into account the heterogeneity across the whole network. Currently, the Bayesian or non-Bayesian network meta-analysis usually assumes a common variance for the random effect estimation; for instance, our analysis of the example data in the previous section assumed that the random effect variances for the comparisons between treatment A and B and between A and C are identical. This assumption effectively reduces the number of parameters to be estimated in the model, rendering it more likely to converge, and saves the computation time. However, it also makes a strong assumption about the distribution of heterogeneity within the network meta-analysis and sometimes may yield ambiguous results. For instance, suppose in a network meta-analysis involving treatment A, B, C, D and E, only one trial that compares A and E was found. If the heterogeneity is large in other parts of the network, the estimated common variance for random effect is likely to be large but the estimated confidence interval for A-E comparison would become greater than that reported by the single trial, even if the evidence within the network is consistent. This is because the confidence interval for A-E comparison reported by the random effect network meta-analysis is the one given under the assumption that A-E comparison has the same degree of heterogeneity as other pairwise comparisons in the network.

The standard random effect network meta-analysis therefore gives rise to a few issues with regard to the assessment of inconsistency between direct and indirect evidence. For treatment contrasts with few head-to-head trials, their confidence interval estimated by traditional pairwise meta-analysis is very likely to be smaller than that given by the random effect network meta-analysis assuming a common random effect variance. Consequently, methods for evaluation of inconsistency between direct and indirect evidence may yield different results under different assumptions with regard to the random effect variance [50, 51].

The UWLS approach provides an alternative way to address the heterogeneity. The parameter *ϕ* in UWLS approach can be interpreted from two perspectives: one is to view *ϕ* as the dispersion parameter to provide a correction to the known with-study standard error ${\sigma}_j^2$. For a common *ϕ*, this can be implemented in most statistical packages. However, if *ϕ* is unique to different treatment contrasts, it will be far more straightforward to fit this type of models in SEM. The other way to interpret *ϕ* is to consider UWLS as a multiplicative random effect model, while the traditional random effect model is additive in the structure of random effect components. In other words, *ϕ* can be viewed as the random effect *τ* ^2^ in Eqs. (6) and (7), where the total variance is *ϕ* + *σ* ^2^, but in UWLS the total variance is *ϕσ* ^2^. Consequently, UWLS is to add *ϕ* into a fixed effect model, making it behave similarly to a random effect model, and a large $\widehat{\phi}$ indicates large treatment effect heterogeneity.

For different pairwise comparisons within the network meta-analysis, we may estimate different *ϕ* in Eq. (8) for different pairwise comparisons. We now extend the UWLS approach to network meta-analysis involving treatment A, B, C, …, K with *p* treatment pairs:

9 $$ \begin{array}{l}{\varDelta}_{c. j}={d}_{AB}+{d}_{AC}+\dots +{d}_{AK}+{v}_{c. j}\\ {}{v}_{c. j} \sim N\left(0,{\phi}_c{\sigma}_{c. j}^2\right)\end{array} $$

The variable Δ_*c* . *j*_ is the treatment contrast *c*, *c* = 1 to *p*, reported by study *j*, *d*_A*k*_, *k* = B to K, are the basic parameters for the comparison between A and *k*, ${\sigma}_{c. j}^2$ is the variance of Δ_*c* . *j*_ and *ϕ*_*c*_ is the variance adjustment factor for treatment contrast *c* within the network meta-analysis.

### UWLS for SEM network meta-analysis

To implement such a model in SEM requires re-arrangement of data. Using the example data for illustration, its UWLS model can be written as:

10 $$ \begin{array}{l}{\varDelta}_{c. j}={d}_{AB}+{d}_{AC}+{v}_{1. j}+{v}_{2. j},\kern0.5em  c=1\  or\ 2\\ {}{v}_{1. j} \sim N\left(0,{\phi}_1{\sigma}_{1. j}^2\right)\\ {}{v}_{2. j} \sim N\left(0,{\phi}_2{\sigma}_{2. j}^2\right)\end{array} $$

where *Δ*_1 . *j*_ is the log odds ratio reported by study *j* that compared treatment A to B and *Δ*_2 . *j*_ the log odds ratio for study *j* that compared treatment A to C; *d*_AB_ is the average treatment difference between A and B; *d*_AC_ is the average treatment difference between A and C; ${\sigma}_{1. j}^2$ is the variance of *Δ*_1 . *j*_; ${\sigma}_{2. j}^2$ is the variance of *Δ*_2 . *j*_; and *ϕ*_1_ and *ϕ*_2_ are the variance adjustment factors for treatment contrasts A-B and A-C, respectively.

We used SEM software package Mplus (version 7.11, Muthen & Muthen, Los Angeles, USA) to undertake all the analyses throughout our study, as Mplus is very flexible in making constraints on parameters estimation. All the data and Mplus codes in this article can be found in the Additional file 1.

## Results

### Contrast-based SEM network meta-analysis for example data

SEM fits simultaneously a group of regression equations, which specify the relationships between observed and latent variables. Latent variables in SEM represent some hidden constructs that cannot be observed or measured directly but have to be estimated from a group of observed (also known as manifest) variables. One special feature of SEM is that the statistical model can be visualized by using a path diagram, and most SEM software packages allow users to draw their path diagrams and undertake the analysis directly. In a path diagram, observed variables are in squares, while latent variables are in circles. A single arrow represents a prediction or causal relationship, e.g. X → Y dipicts that X predicts Y or X causes Y. A double arrow represents a correlation or covariance, e.g. X ↔ Y depicts that X and Y are correlated. Results show that the log odds ratio for treatment A and B is −0.485 (95% Confidence Interval [CI]: −0.717 to −0.254) and for A and C is −0.600 (95% CI: -0.932 to −0.268).

**Fig. 1:**
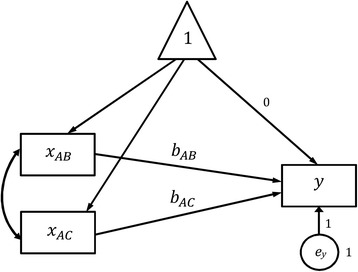
Path diagram for the fixed effect network meta-analysis model

**Fig. 2:**
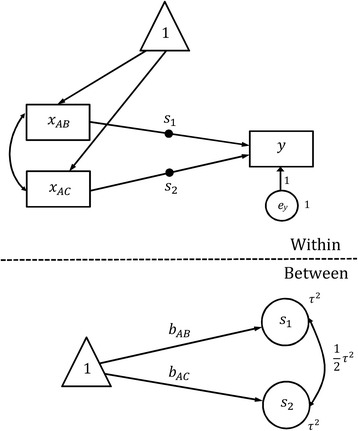
Path diagram for the random effect network meta-analysis model

Figures 1 and 2 show the path diagrams for Eqs. (6) and (7) with fixed and random effects, respectively, demonstrating how to use the multilevel SEM to undertake the random effect network meta-analysis for example data. In the level-1 model (the Within-level in Fig. 2), *y* is the transformed log odds ratio, and *x*_AB_ and *x*_AC_ are the transformed *t*_AB_ and *t*_AC_, respectively. The filled circle on the arrow from *x*_AB_ to *y* represents random slope that is referred to as *s*_1_ in the level-2 model (the Between-level in Fig. 2). The filled circle on the arrow from *x*_AC_ to *y* represents random slope that is referred to as *s*_2_ in the level-2 model. The variance of *s*_1_ and *s*_2_ is *τ* ^2^ in Eq. (7), and their covariance is constrained to be $\frac{1}{2}{\tau}^2$. Note that in Fig. 2 there is no random intercept, and the intercept of *y* is fixed at 0. The arrows from the variable in triangle to *s*_1_ and *s*_2_ indicate that the means of *s*_1_ and *s*_2_ are estimated, which give rise to *β*_*AB*_ and *β*_*AC*_ in Eq. (7). The variance of the residual error term *e*_*y*_ is fixed at unity. Results show that the log odds ratio for treatment A and B is −0.585 (95% CI: -1.087 to −0.082) and for A and C is −0.711 (95% CI: -1.438 to 0.016).

### UWLS for SEM network meta-analysis for example data

**Fig. 3:**
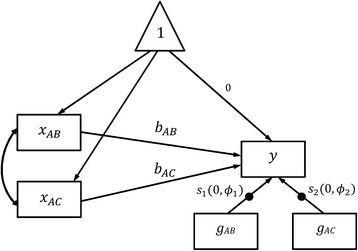
Path diagram for the unrestricted weighted least squares (UWLS) network meta-analysis model

To estimate UWLS model with common variance adjustment factors *ϕ*_1_ = *ϕ*_2_ in Eq. (10), we only need to remove the constraint on the variance of *e*_*y*_ in the fixed effect network meta-analysis model shown in Fig. 1. Table 1 showed results from Mplus for the fixed effect, random effect, and the two UWLS models. Results from Mplus show that *ϕ* is 3.563, and the log odds ratio for treatment A and B is −0.485 (95% CI: -0.922 to −0.049) and for A and C is −0.600 (95% CI: -1.227 to 0.027). The point estimates are identical to those in the fixed effect model but the confidence intervals are greater. To estimate the UWLS model with unique variance adjustment factors in Eq. (10), we need to create two residual error terms for *y*: one for studies reporting treatment contrasts A-B and the other for those reporting treatment contrast A-C. Figure 3 shows the path diagram, where *y* is the transformed log odds ratios and is regressed on *x*_AB_ and *x*_AC_, which are the transformed variables *t*_AB_ and *t*_AC_, respectively. Variables *g*_AB_ and *g*_AC_ are dummy variables for studies reporting treatment contrasts A-B and A-C, respectively. The filled circle on the arrow from *g*_AB_ to *y* represents random slope that is labelled as *s*_1_, and the filled circle on the arrow from *g*_AC_ to *y* represents random slope that is labelled as *s*_2_. The means of *s*_1_ and *s*_2_ are fixed at zero, and the variances of *s*_1_ and *s*_2_ are *ϕ*_1_ and *ϕ*_2_ in Eq. 8, respectively, with their covariance being fixed at zero. In this model, the residual error for *y* is split into two independent random variables *s*_1_ and *s*_2_, and their variances are estimated separately. Results from Mplus show that the log odds ratio for treatment A and B is −0.484 (95% CI: -0.958 to −0.010) and for A and C is −0.600 (95% CI: -1.075 to −0.125). The point estimates are almost identical to those given by the fixed effect model, but the confidence intervals are greater. Compared to the confidence intervals reported by the UWLS model with common *ϕ*, the confidence interval for *x*_AB_ is greater but that for *x*_AC_ is smaller. This is because the variance adjustment factors *ϕ*_1_ and *ϕ*_2_ are 4.288 and 2.031, respectively, indicating a greater degree of heterogeneity within A-B head-to-head trials. This is consistent with results from the traditional pairwise meta-analyses in which the degree of heterogeneity in studies reporting treatment contrast A-B is greater than that of studies reporting contrast A-C.

**Table 1:** Results of four SEM models for the example data

	Fixed effect model	Random effect model	UWLS model with common ϕ	UWLS model with unique ϕ
Fixed effect coefficients				
bAB	−0.485 (−0.717 to −0.254)	−0.585 (−1.087 to −0.082)	−0.485 (−0.922 to −0.049)	−0.485 (−0.958 to −0.010)
bAC	−0.600 (−0.932 to −0.268)	−0.711 (−1.438 to 0.016)	−0.600 (−1.227 to 0.027)	−0.600 (−1.075 to −0.125)
Random effect coefficients				
*τ* ^2^		0.877		
ϕ				
AB			3.563	4.288
AC			3.563	2.031

## Discussion

In this article, we demonstrate how to undertake network meta-analysis within the statistical framework of structural equation modeling. While issues such as the evaluation of inconsistency between direct and indirect evidence are important and can be integrated into SEM framework, it is beyond the scope of the present study to discuss these issues. Standard statistical software packages for generalized linear mixed modeling may be used to analyze the fixed and random effect models discussed in this article, but SEM software packages are more flexible in specifying complex covariance structure and imposing constraints on parameter estimation. Our results are very close to those reported in previous publications using the command mvmeta for the statistical software package Stata [25, 52]. The estimated random effect variance *τ* ^2^ is 0.877, which is slightly smaller than that given by mvmeta in Stata. Mplus only implements maximum likelihood estimation rather than restricted maximum likelihood estimation [53], but maximum likelihood estimation tends to under-estimate the variance component in multilevel models [43]. However, metaSEM package in R has implemented restricted maximum likelihood estimation and can be used to fit multivariate meta-analysis and network meta-analysis [43, 53].

It is quite straightforward to implement UWLS approach to network meta-analysis with heteroscedastic errors in SEM. In the UWLS approach, the between and within-study heterogeneities are considered multiplicative (*ϕσ* ^2^), and this is different from the traditional random effect model, where they are considered additive (*σ* ^2^ + *τ* ^2^). The additive random effect assumes that the between and within-study heterogeneities are independent, while the multiplicative random effect assumes that the between and within-study heterogeneities are related. As within-study heterogeneity *σ* ^2^ is a known quantity, it can then be viewed as the weight for the between-study heterogeneity *ϕ*. Two recent studies compare the performance of additive or multiplicative heterogeneity in traditional pairwise meta-analyses and found that results of these two models tend to agree but multiplicative model produces narrower confidence intervals [48, 49]. Further research is warranted to compare their performance in network meta-analyses.

## Conclusion

SEM provides a useful framework for univariate and multivariate meta-analysis, and its potential as a powerful tool for advanced meta-analysis is still to be explored.

## Additional file

Additional file 1: The dataset and Mplus scripts used for statistical analysis. (DOCX 110 kb)

## Abbreviations

SEM: Structural equation modeling
UWLS: Unrestricted weighted least squares
