# Supplementary material for: Using structural equation modeling for network meta-analysis
Source: BMC Med Res Methodol. 2017 Jul 14;17:104. doi: 10.1186/s12874-017-0390-9 (PMC5512972; doi:10.1186/s12874-017-0390-9)

**Online supplementary materials for “Using Structural Equation Modeling for Network Meta-analysis**

Yu-Kang Tu ^1^

Yun-Chun Wu ^1^

^1^ Department of Public Health and Institute of Epidemiology & Preventive Medicine, College of Public Health, National Taiwan University, Taipei, Taiwan

Table 1. Sclerotherapy data

| Study | Trt1 | Trt 2 | r1 | n1 | r2 | n2 | dAA | dAB | dAC | lnOR | var(lnRR) |
| --- | --- | --- | --- | --- | --- | --- | --- | --- | --- | --- | --- |
| 1 | A | C | 13 | 41 | 2 | 43 | -1 | 0 | 1 | -2.25 | 0.64 |
| 1 | A | B | 13 | 41 | 9 | 42 | -1 | 1 | 0 | -0.53 | 0.25 |
| 2 | A | C | 13 | 72 | 12 | 68 | -1 | 0 | 1 | -0.03 | 0.20 |
| 2 | A | B | 13 | 72 | 13 | 73 | -1 | 1 | 0 | -0.02 | 0.19 |
| 3 | A | C | 4 | 16 | 4 | 20 | -1 | 0 | 1 | -0.29 | 0.65 |
| 4 | A | C | 30 | 111 | 20 | 116 | -1 | 0 | 1 | -0.58 | 0.11 |
| 5 | A | C | 11 | 49 | 1 | 30 | -1 | 0 | 1 | -2.13 | 1.15 |
| 6 | A | C | 10 | 53 | 7 | 53 | -1 | 0 | 1 | -0.42 | 0.29 |
| 7 | A | C | 31 | 89 | 18 | 85 | -1 | 0 | 1 | -0.69 | 0.12 |
| 8 | A | C | 11 | 51 | 2 | 51 | -1 | 0 | 1 | -1.91 | 0.64 |
| 9 | A | C | 2 | 25 | 8 | 23 | -1 | 0 | 1 | 1.81 | 0.74 |
| 10 | A | B | 0 | 19 | 4 | 18 | -1 | 1 | 0 | 2.49 | 2.34 |
| 11 | A | B | 22 | 36 | 3 | 35 | -1 | 1 | 0 | -2.82 | 0.48 |
| 12 | A | B | 30 | 53 | 5 | 56 | -1 | 1 | 0 | -2.59 | 0.30 |
| 13 | A | B | 6 | 18 | 5 | 16 | -1 | 1 | 0 | -0.10 | 0.54 |
| 14 | A | B | 9 | 22 | 3 | 23 | -1 | 1 | 0 | -1.53 | 0.57 |
| 15 | A | B | 31 | 46 | 11 | 49 | -1 | 1 | 0 | -1.97 | 0.22 |
| 16 | A | B | 9 | 60 | 19 | 53 | -1 | 1 | 0 | 1.15 | 0.21 |
| 17 | A | B | 26 | 60 | 17 | 53 | -1 | 1 | 0 | -0.48 | 0.15 |
| 18 | A | B | 29 | 69 | 10 | 71 | -1 | 1 | 0 | -1.49 | 0.18 |
| 19 | A | B | 14 | 41 | 12 | 41 | -1 | 1 | 0 | -0.23 | 0.23 |
| 20 | A | B | 3 | 20 | 0 | 21 | -1 | 1 | 0 | -2.15 | 2.39 |
| 21 | A | B | 14 | 35 | 13 | 33 | -1 | 1 | 0 | -0.03 | 0.25 |
| 22 | A | B | 23 | 138 | 31 | 143 | -1 | 1 | 0 | 0.32 | 0.09 |
| 23 | A | B | 19 | 51 | 20 | 55 | -1 | 1 | 0 | -0.04 | 0.16 |
| 24 | A | B | 12 | 16 | 3 | 13 | -1 | 1 | 0 | -2.30 | 0.77 |
| 25 | A | B | 5 | 28 | 3 | 21 | -1 | 1 | 0 | -0.27 | 0.63 |
| 26 | A | B | 2 | 24 | 6 | 22 | -1 | 1 | 0 | 1.42 | 0.77 |

**R code for transforming original data into the data for Mplus analysis**

#define the variance covariance matrix

V<-diag(trial$var)

V[1,2]<-0.113

V[2,1]<-0.113

V[3,4]<-0.094

V[4,3]<-0.094

#use Cholesky decomposition method to decompose the maxtrix

# chol gives the upper triangular matrix

L<-chol(solve(V))

#y transformation

y<-trial$lnor

y.w<-L%*%y

#x transformation

x<-as.matrix(trial[,8:10])

x.w<-L%*%X

Table 2: Sclerotherapy data for Mplus analysis

| study | y | xaa | xab | xac | gab | gac |
| --- | --- | --- | --- | --- | --- | --- |
| 1 | -2.63252 | -0.72481 | -0.58066 | 1.30547 | 0 | 1 |
| 1 | -1.05554 | -1.98399 | 1.98399 | 0 | 1 | 0 |
| 2 | -0.05052 | -1.29627 | -1.30374 | 2.60001 | 0 | 1 |
| 2 | -0.03882 | -2.30964 | 2.30964 | 0 | 1 | 0 |
| 3 | -0.35797 | -1.24434 | 0 | 1.24434 | 0 | 1 |
| 4 | -1.76642 | -3.07009 | 0 | 3.07009 | 0 | 1 |
| 5 | -1.98253 | -0.93181 | 0 | 0.93181 | 0 | 1 |
| 6 | -0.7905 | -1.86387 | 0 | 1.86387 | 0 | 1 |
| 7 | -1.98586 | -2.88699 | 0 | 2.88699 | 0 | 1 |
| 8 | -2.3915 | -1.25361 | 0 | 1.25361 | 0 | 1 |
| 9 | 2.11538 | -1.16631 | 0 | 1.16631 | 0 | 1 |
| 10 | 1.62919 | -0.65338 | 0.65338 | 0 | 1 | 0 |
| 11 | -4.06283 | -1.44118 | 1.44118 | 0 | 1 | 0 |
| 12 | -4.75364 | -1.83674 | 1.83674 | 0 | 1 | 0 |
| 13 | -0.12959 | -1.35968 | 1.35968 | 0 | 1 | 0 |
| 14 | -2.02331 | -1.32295 | 1.32295 | 0 | 1 | 0 |
| 15 | -4.22789 | -2.15091 | 2.15091 | 0 | 1 | 0 |
| 16 | 2.49897 | -2.16797 | 2.16797 | 0 | 1 | 0 |
| 17 | -1.22647 | -2.54432 | 2.54432 | 0 | 1 | 0 |
| 18 | -3.54504 | -2.3845 | 2.3845 | 0 | 1 | 0 |
| 19 | -0.47428 | -2.10221 | 2.10221 | 0 | 1 | 0 |
| 20 | -1.39204 | -0.64693 | 0.64693 | 0 | 1 | 0 |
| 21 | -0.05105 | -2.01631 | 2.01631 | 0 | 1 | 0 |
| 22 | 1.06342 | -3.27279 | 3.27279 | 0 | 1 | 0 |
| 23 | -0.09507 | -2.48105 | 2.48105 | 0 | 1 | 0 |
| 24 | -2.62974 | -1.14208 | 1.14208 | 0 | 1 | 0 |
| 25 | -0.33413 | -1.25752 | 1.25752 | 0 | 1 | 0 |
| 26 | 1.61007 | -1.1362 | 1.1362 | 0 | 1 | 0 |

1.1 Mplus codes for fixed effect network meta-analysis:

Title:

Sclerotherapy for fixed effect trial level analysis

Data:

File is sclerochol.dat ;

Variable:

Names are

study y xaa xab xac gab gac;

Missing are all (-9999) ;

Usevariables are y xab xac ;

Analysis:

Estimator = ML;

Model:

y ON xab xac;

[xab xac y@0];

y@1;

Output: cinterval

1.2 Model Results for fixed effect network meta-analysis:

MODEL RESULTS

Two-Tailed

Estimate S.E. Est./S.E. P-Value

Y ON

XAB -0.485 0.118 -4.111 0.000

XAC -0.600 0.169 -3.541 0.000

XAC WITH

XAB -0.821 0.254 -3.229 0.001

Means

XAB 1.154 0.211 5.472 0.000

XAC 0.583 0.180 3.230 0.001

Intercepts

Y 0.000 0.000 999.000 999.000

Variances

XAB 1.246 0.333 3.742 0.000

XAC 0.912 0.244 3.742 0.000

Residual Variances

Y 1.000 0.000 999.000 999.000


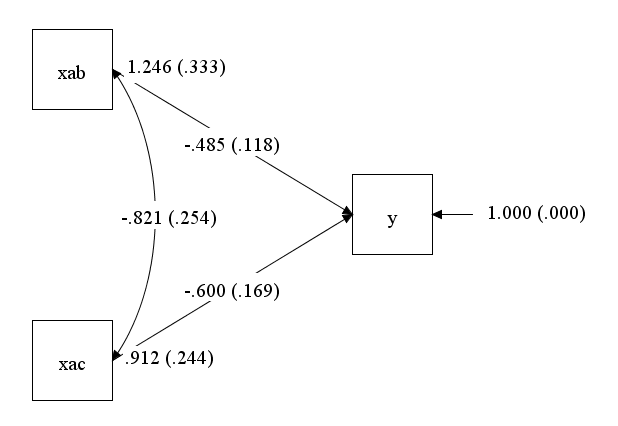


2.1 Mplus codes for random effect network meta-analysis:

Title:

Sclerotherapy for random effect trial level analysis

Data:

File is G:\sclerochol.dat ;

Variable:

Names are

study y xaa xab xac gab gac;

Missing are all (-9999) ;

Usevariables are y xab xac ;

cluster = study;

within = xab xac;

Analysis:

Type = twolevel random;

Estimator = ML;

Model:

%within%

! int only has random but not fixed effects

S1|y ON xab;

S2|y ON xac;

y@1;

%between%

y@0;

s1 (v1)*1;

s2 (v1)*1;

s1 WITH s2 (cv1);

[y@0 s1 s2];

Model constraint:

cv1 = 0.5*v1;

Output: cinterval

2.2 Model Results for random effect network meta-analysis:

MODEL RESULTS

Two-Tailed

Estimate S.E. Est./S.E. P-Value

Within Level

Residual Variances

Y 1.000 0.000 999.000 999.000

Between Level

S1 WITH

S2 0.439 0.186 2.363 0.018

Means

Y 0.000 0.000 999.000 999.000

S1 -0.585 0.256 -2.280 0.023

S2 -0.711 0.371 -1.916 0.055

Variances

Y 0.000 0.000 999.000 999.000

S1 0.877 0.371 2.363 0.018

S2 0.877 0.371 2.363 0.018

Note that: Mplus does not provide path diagram for random effect models

3.1 Mplus codes for unrestricted weighted least square network meta-analysis with common $\phi$:

Title:

Sclerotherapy for unrestricted weighted least square trial level analysis

Data:

File is sclerochol.dat ;

Variable:

Names are

study y xaa xab xac gab gac;

Missing are all (-9999) ;

Usevariables are y xab xac ;

Analysis:

Estimator = ML;

Model:

y ON xab xac;

[xab xac y@0];

Output: cinterval

3.2 Model Results for unrestricted weighted least square network meta-analysis with common $\phi$:

MODEL RESULTS

Two-Tailed

Estimate S.E. Est./S.E. P-Value

Y ON

XAB -0.485 0.223 -2.178 0.029

XAC -0.600 0.320 -1.876 0.061

XAC WITH

XAB -0.821 0.254 -3.229 0.001

Means

XAB 1.154 0.211 5.472 0.000

XAC 0.583 0.180 3.230 0.001

Intercepts

Y 0.000 0.000 999.000 999.000

Variances

XAB 1.246 0.333 3.742 0.000

XAC 0.912 0.244 3.742 0.000

Residual Variances

Y 3.563 0.952 3.742 0.000


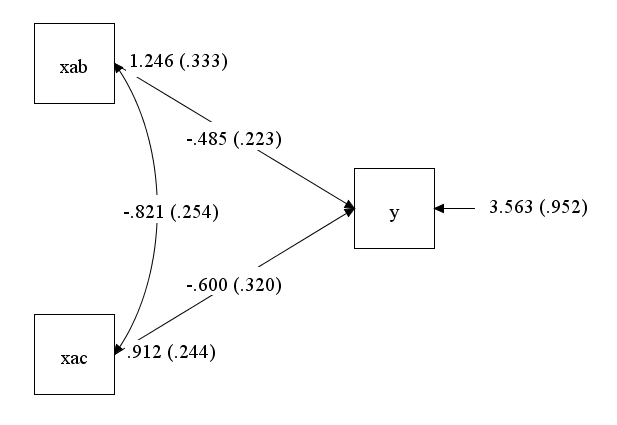


4.1 Mplus codes for unrestricted weighted least square network meta-analysis with unique $\phi$:

Title:

Sclerotherapy for unrestricted weighted least square trial level analysis

Data:

File is sclerochol.dat ;

Variable:

Names are

study y xaa xab xac gab gac;

Missing are all (-9999) ;

Usevariables are y xab xac gab gac;

!grouping is g (0 = AC 1 = AB);

Analysis:

Type = random;

Estimator = ML;

MCONVERGENCE = 0.000001;

Model:

y ON xab xac;

s1| y ON gab;

s2| y ON gac;

[y@0 s1@0 s2@0 xab xac];

y@0;

s1 WITH s2@0;

Output: cinterval

4.2 Model Results for unrestricted weighted least square network meta-analysis with unique $\phi$:

MODEL RESULTS

Two-Tailed

Estimate S.E. Est./S.E. P-Value

Y ON

XAB -0.484 0.242 -2.002 0.045

XAC -0.600 0.243 -2.474 0.013

S1 WITH

S2 0.000 0.000 999.000 999.000

XAC WITH

XAB -0.821 0.254 -3.229 0.001

Means

XAB 1.154 0.211 5.472 0.000

XAC 0.583 0.180 3.230 0.001

S1 0.000 0.000 999.000 999.000

S2 0.000 0.000 999.000 999.000

Intercepts

Y 0.000 0.000 999.000 999.000

Variances

XAB 1.246 0.333 3.742 0.000

XAC 0.912 0.244 3.741 0.000

S1 4.288 1.391 3.082 0.002

S2 2.031 0.957 2.121 0.034

Residual Variances

Y 0.000 0.000 999.000 999.000


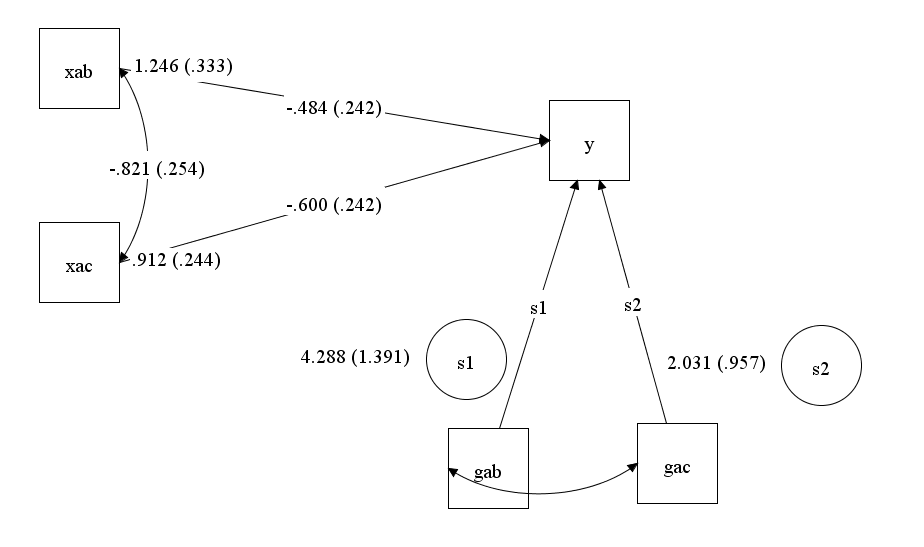

Supplement: Additional file 1: — The dataset and Mplus scripts used for statistical analysis. (DOCX 110 kb) [file 12874_2017_390_MOESM1_ESM.docx]
